# A threat to our sustainable future is hiding on our winter roads

**DOI:** 10.1016/j.eehl.2025.100204

**Published:** 2025-11-24

**Authors:** Jingzhe Wang, Xuankai Ma, Nan Xu

**Affiliations:** a School of Artificial Intelligence, Shenzhen Polytechnic University, Shenzhen 518055, China; b Urumqi Urban Institute of Geotechnical Investigation Surveying and Mapping, Urumqi 830000, China; c Key Laboratory for Geo-Environmental Monitoring of Great Bay Area, Ministry of Natural Resources & Guangdong Key Laboratory of Urban Informatics, Shenzhen University, Shenzhen 518060, China; d School of Architecture and Urban Planning, Shenzhen University, Shenzhen 518060, China

The annual application of millions of tons of deicing salt creates a critical conflict between public safety and environmental security [1]. While essential for winter travel, deicing salt operates as a cross-media pollutant. Its runoff infiltrates soil and enters waterways, degrading agricultural lands, diminishing crop yields, and impairing the soil's vital capacity for carbon storage [2]. This escalating damage directly undermines global progress on food security (SDG 2) and terrestrial ecosystems (SDG 15) [3].

Such damage persists due to a fundamentally flawed management paradigm that traps us in a costly cycle of treating symptoms rather than the cause, leading to escalating remediation burdens and potentially irreversible outcomes, such as the permanent loss of soil fertility or the contamination of drinking water sources. This failure is threefold. Spatially, its focus is myopic, confined to the road surface while ignoring the interconnected watershed. Operationally, its timing is reactive, applying materials only after hazardous ice has formed. Strategically, its planning is blind, lacking the foresight to anticipate and prevent future pollution hotspots.

We propose a framework that systematically corrects these failings. First, management must adopt a holistic, watershed-scale stewardship model [4]. Next, operations must shift from reactive to proactive, prioritizing anti-icing with brine and/or pre-wet salt to prevent ice bonding and cut salt use substantially [5,6]. Finally, planning must overcome strategic blindness by employing coupled hydrological-terrestrial models to predict high-risk zones, guiding smarter, preventative interventions before contamination occurs [7].

Translating this framework into practice requires a new synergy between science and policy. We call on researchers to quantify the full life-cycle costs of current deicing practices, and for policymakers to champion legal frameworks that incentivize smarter practices. Integrating our approach to winter safety with ecological stewardship is not a choice, but a necessity for a sustainable future.

## CRediT authorship contribution statement

**Jingzhe Wang:** Conceptualization, Formal analysis, Funding acquisition, Project administration, Writing – original draft. **Xuankai Ma:** Conceptualization, Investigation, Writing – original draft. **Nan Xu:** Funding acquisition, Investigation, Project administration, Writing – original draft.

## Declaration of competing interest

The authors declare no competing interests.
